# BMP-2 induced *Dspp* transcription is mediated by Dlx3/Osx signaling pathway in odontoblasts

**DOI:** 10.1038/s41598-017-10908-8

**Published:** 2017-09-07

**Authors:** Guobin Yang, Guohua Yuan, Mary MacDougall, Chen Zhi, Shuo Chen

**Affiliations:** 1 The State Key Laboratory Breeding Base of Basic Science of Stomatology & Key Laboratory of Oral Biomedicine Ministry of Education, School and Hospital of Stomatology, Wuhan University, Wuhan, 430079 China; 2 Department of Development Dentistry, Dental School, The University of Texas Health Science Center at San Antonio, San Antonio, 78229 USA; 3 Department of Oral Maxillofacial Surgery, Institute of Oral Health Research, School of Dentistry, University of Alabama at Birmingham, Birmingham, 35294-0007 Alabama USA

## Abstract

Dentin sialophosphoprotein (*Dspp*) as a differentiation marker of odontoblasts is regulated by BMP-2. However, the intimate mechanism is still unknown. Transcription factors Dlx3 and Osx are essential for odontoblasts differentiation. We hypothesized that BMP-2 regulation of *Dspp* transcription was mediated by Dlx3 and/or Osx in odontoblasts. In the present investigation, we found that BMP-2 stimulated expression and nuclear translocation of Dlx3 and Osx in odontoblasts both *in vitro* and *in vivo*. Osx was a downstream target of Dlx3 and both of them stimulated Dsp expression. Both Dlx3 and Osx were able to activate *Dspp* promoter from nucleotides (nt) −318 to +54 by transfections of luciferase reports containing different lengths of mouse *Dspp* promoters. The binding of Dlx3 and Osx with nt −318 to +54 of *Dspp* promoter was verified by chromatin immunoprecipitation *in vivo*. Two Dlx3 binding sites and one Osx binding site on *Dspp* promoter were found by EMSA. Furthermore, the exact biological function of these binding sites was confirmed by site-directed mutagenesis. At last, the protein-protein interaction between Dlx3 and Osx in odontoblasts was detected by co-immunoprecipitation. In conclusion, in this study we found a novel signaling pathway in which BMP-2 activates *Dspp* gene transcription via Dlx3/Osx pathway.

## Introduction

The dentin sialophosphoprotein (*Dspp*) gene encodes the major non-collagenous protein in dentin matrix, which is expressed predominantly in preodontoblasts and odontoblasts and transiently in preameloblasts, and at low levels in osteoblasts^1–4^. DSPP is a highly phosphorylated protein that is cleaved into 3 proteins, dentin sialoprotein (DSP), dentin glycoprotein (DGP), and dentin phosphoprotein (DPP), immediately after secretion^5, 6^. DSP is the N-terminal portion of DSPP and is a 95-kDa glycoprotein that was first identified within the extracellular matrix of dentin^5^. DGP was identified as an 81-amino acid segment between DSP and DPP^6^. DPP is rich in aspartic acid and phosphoserine and binds to calcium. Therefore, DPP is strongly associated with mineral phase of dentin, acting as an important initiator and modulator of dentin apatite crystal formation, which makes DPP a specific marker for terminal differentiated odontoblasts^5, 7, 8^. Several human and mouse genetic studies have indicated that *Dspp* is important for dentin mineralization. Mutations of human *DSPP* gene are associated with human hereditary disorders such as dentinogenesis imperfecta type II (DGI-II), type III (DGI-III), and dentin dysplasia type II (DD-II)^9–13^. *Dspp* knockout mice exhibit dentin mineralization defects that are very similar to human DGI-III as well as impaired cranial bone development^14, 15^. These studies demonstrate that *Dspp* plays a crucial role in tooth development and mineralization, in particular dentinogenesis.

Bone morphogenetic proteins (BMPs) are structurally related to the transforming growth factor β (TGF-β) superfamily. Among the BMP family members, BMP-2 plays important roles during odontogenic differentiation^16^. *Bmp-2* is initially expressed in the dental epithelial cells at embryonic day 12.5 (E12.5), then shifts to the dental mesenchymal papilla and is involved in specifying the fate of the dental mesenchymal cells at later stage of tooth development^17, 18^. During the late stage of tooth development, *Bmp-2* becomes more intense in the terminal differentiated odontoblasts and regulates the differentiation of odontoblasts^16, 19, 20^. The odontoblasts do not mature properly and fail to form proper dentin with normal dentinal tubules and activate terminal differentiation with the deletion of the *Bmp*-2 gene in odontoblasts^16^. Studies have shown that *Dspp* in odontoblasts is regulated by BMP signaling, especially BMP-2, *in vitro* and *in vivo* ^3, 21, 22^. Beads socked in human recombinant BMP-2 induce the mRNA expression of *Dspp*, after implantation onto dental papilla in organ culture^23^. *Dspp* expression was reduced in the *Bmp-2* knock out mice^16^. Previously we also have found that BMP-2 up-regulates *Dspp* transcription through its regulatory region in mouse preodontoblast cells^3^. However, the intimate molecular mechanisms of BMP-2 regulating *Dspp* transcription in preodontoblasts or odontoblasts have not been completely understood.

Transcription factors Dlx3 and Osx (or Sp7, Osterix) are essential for osteoblast and odontoblast differentiation^24–26^. Both Dlx3 and Osx are suggested as downstream targets of BMP-2 signaling in osteogenic cells^27, 28^. Although both odontoblasts and osteoblasts originate from mesenchymal cells and share many physical similarities, the molecular mechanisms regulating odontoblasts differentiation are different from osteoblasts. For example, BMPs are able to stimulate expression of Runx2 in osteoblastic cells but not in odontoblasts^2, 29^. Whether Dlx3 and Osx mediate the BMP-2 induced Dspp expression in odontoblasts is still not known. During tooth morphogenesis, *Dlx3* mRNA is initially expressed in the dental epithelium, and is later expressed in both the dental epithelium and the dental mesenchyme^25^. Mutations of *DLX3* in human cause Tricho-Dento-Osseous (TDO) syndrome, which is an autosomal dominant disorder characterized by defects in ectodermal derivatives such as hair (kinky hair), teeth (enamel hypoplasia and taurodontism) and bone (increased bone density in cranium and long bones)^30–32^. Deletion of *Dlx3* in neural crest, from which dental mesenchyme is derived, leads to severe dentin hypoplasia and dysplasia and remarkably down-regulated *Dspp* expression^33^. During tooth development, *Osx* expression was initiated at dental papilla, and remained highly expressed in differentiating odontoblasts at later stages^21^. Previously, we found overexpression of Dlx3 or Osx in odontogenic cells induced cell differentiation and *Dspp* expression^34, 35^,while knock-down of Osx caused the down-regulation of Dsp expression^36^.

Based on these biological functions of Dlx3 and Osx during odontoblasts differentiation and tooth development, we hypothesized that BMP-2 regulation of *Dspp* transcription was mediated by Dlx3 and/or Osx signaling pathway in odontoblasts.

## Results

### BMP-2 stimulates expression and nuclear translocation of both Dlx3 and Osx in odontoblast cells

**Figure 1:**
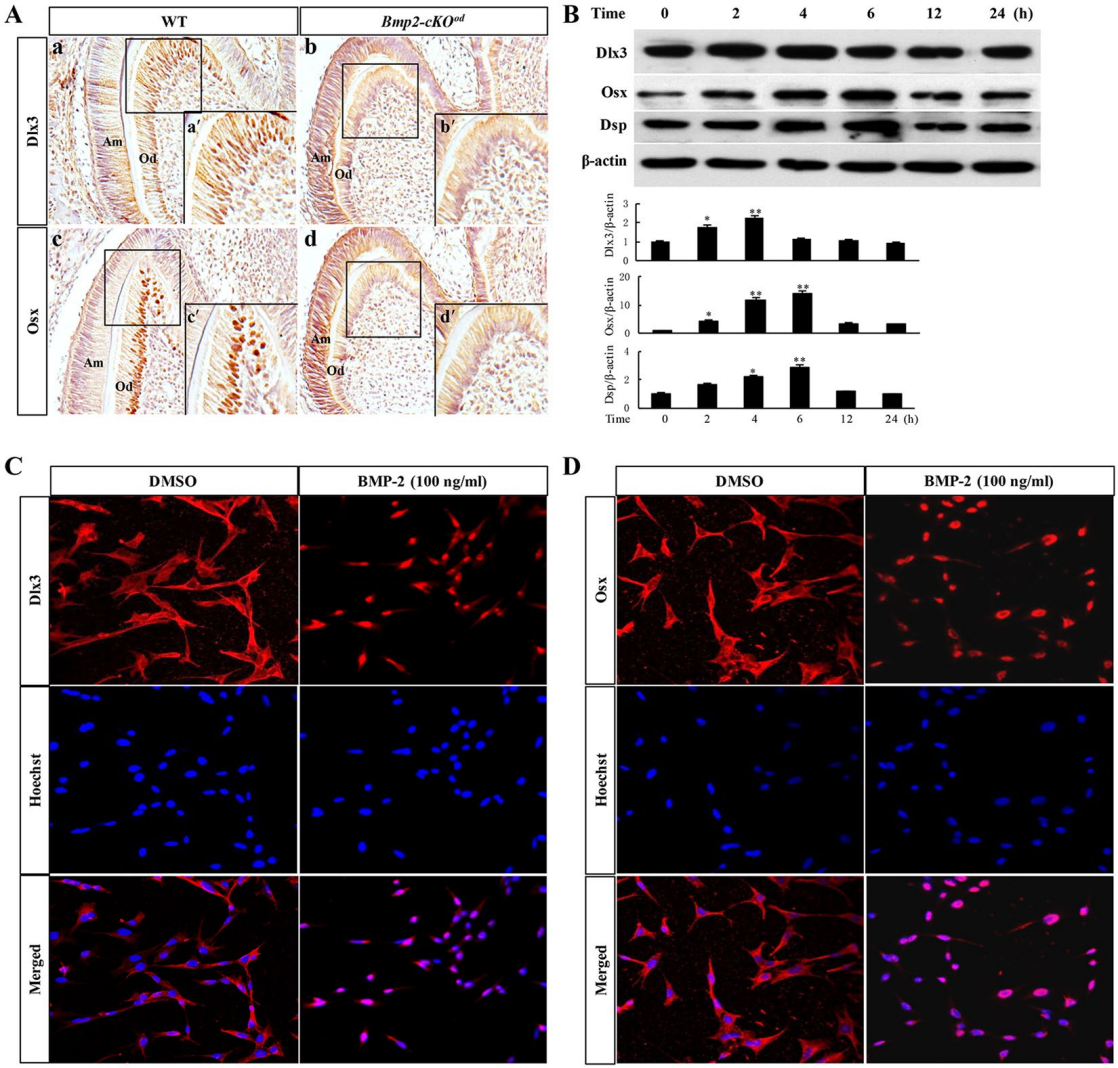
Effect of BMP-2 on expression and nuclear translocation of Dlx3, Osx in mouse preodontoblasts or odontoblasts. (**A**) Immunohistochemistry showed the expressions of Dlx3 (a and b) and Osx (c and d) in tooth germs of wild type (a and c) and *Bmp2-cKO* ^*od*^ (b and d) mice at P3. Inserted figures (a′,b′,c′ and d′) represented higher magnification of the squares. (**B**) The expressions of Dlx3, Osx, and Dsp were assessed by Western blot in MD10-F2 cells with recombinant BMP-2 treatment for 0–24 h. Lower panel showed the quantified data assessed by ImageJ software. *P < 0.05; **P < 0.01; compared with 0 h. (**C**) and (**D)** Immunofluorescence staining showed the nuclear translocation of Dlx3 and Osx in MD10-F2 cells with recombinant BMP-2 stimulation for 2 h. Uncropped images of blots were shown in Supplementary Fig. S2.

We first detected the expressions of Dlx3 and Osx in odontoblasts of first molars in *Bmp2-cKO°* ^*d*^ mice. With the knock-out of *Bmp2* gene in odontoblasts (showed by *in situ* hybridization in Supplementary Fig. S1), the odontoblasts of first molars in *Bmp2-cKO°* ^*d*^ mice lost their differentiation and polarization, and accompanied with dramatically reduced expressions of Dlx3 and Osx compared with wild type mice, in which high expression of Dlx3 and Osx was seen in the nuclei of odontoblasts (Fig. 1A). Then we examined whether BMP-2 is able to induce expressions of Dlx3 and Osx in mouse pre-odontoblasts (MD10-F2). Our previous study indicated that BMP-2 (100 ng/ml) was sufficient to induce *Dspp* expression in MD10-F2 cells^3^. Therefore, a final concentration of 100 ng/ml of BMP-2 recombinant protein was added in the medium to stimulate MD10-F2 cells, and expressions of Dlx3, Osx, and Dsp were measured by Western blot. As illustrated in Fig. 1B, expression of Dlx3 was increased and reached the maximum level at 2–4 hours after BMP-2 treatment. Meanwhile, the expressions of Osx and Dsp were also up-regulated and reached the maximum level at 4–6 hours after BMP-2 treatment. All these *in vivo* and *in vitro* data indicated both Dlx3 and Osx are downstream targets of BMP-2 in odontoblasts. As transcription factors, Dlx3 and Osx, activated by BMP-2 should paly functions in nucleus. Then we detected the subcellular localization of Dlx3 and Osx after BMP-2 induction using immunofluorescence (Fig. 1C,D). In untreated cells, Dlx3 and Osx staining appeared mainly in the cytoplasm and weakly in nucleus. After induction with BMP-2, the staining of Dlx3 and Osx mainly localized into nucleus, compared to that in the untreated cells. These results suggested that BMP-2 not only stimulated expression of Dlx3, Osx and Dsp, but also induced nuclear translocation of Dlx3 and Osx *in vitro* and *in vivo*.

### Osx is a downstream target of Dlx3 and both of them stimulate Dsp expression in MD10-F2 cells

**Figure 2:**
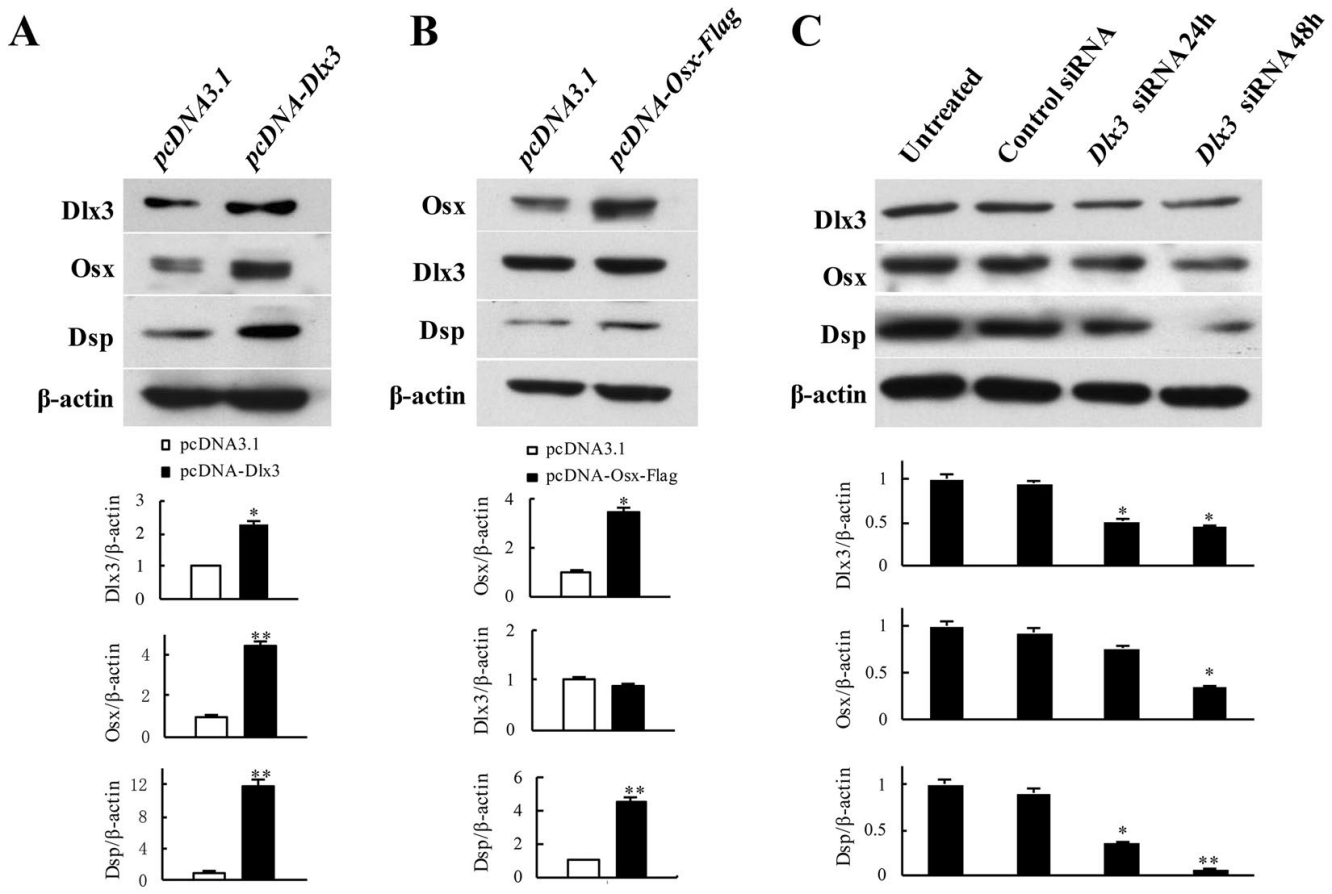
Effect of Dlx3 and Osx on Dsp expression in MD10-F2. (**A**) Cells were transfected with either *pcDNA3*.*1* or *pcDNA-Dlx3* for 48 h. (**B**) Cells were transfected with either *pcDNA3*.*1* or *pcDNA-Osx-Flag* for 48 h. (**C**) Cells were transfected with either negative control siRNA or siRNA against mouse *Dlx3* for 24 h or 48 h. The expression of Dlx3, Osx, Dsp and β-actin were detected by Western blot. Lower panels showed the quantified data assessed by ImageJ software. *P < 0.05; **P < 0.01; compared with cells transfected with *pcDNA 3*.*1* or treated with DMSO. Uncropped images of blots were shown in Supplementary Fig. S3.

It was reported that Osx is an explicit downstream target of Dlx5 in osteoblast-lineage cells^37^. Although Dlx5 and Dlx3 belong to the Distaless (Dlx) gene family, it is yet not known if Dlx3 is able to regulate Osx in odontoblasts. We therefore tested the relationship between Dlx3 and Osx in MD10-F2 cells. The results showed that overexpression of Dlx3 was able to induce Osx and Dsp expression (Fig. 2A), whereas overexpression of Osx only induced Dsp expression but did not influence the expression of Dlx3 (Fig. 2B). To further confirm these results, we knocked down the endogenous Dlx3 expression using *Dlx3*-targeted siRNA (Fig. 2C). Transfection of *Dlx3* siRNA for 48 hours reduced Dlx3 expression more than 50% in MD10-F2 cells. At the same time, Osx and Dsp expression was reduced to ~40% and ~90%, respectively, compared with their expression in cells transfection with control siRNA. Thus, Osx was a downstream target of Dlx3, and both of them were able to stimulate Dsp expression in MD10-F2 cells.

### Dlx3 and Osx stimulate Dspp promoter activity

**Figure 3:**
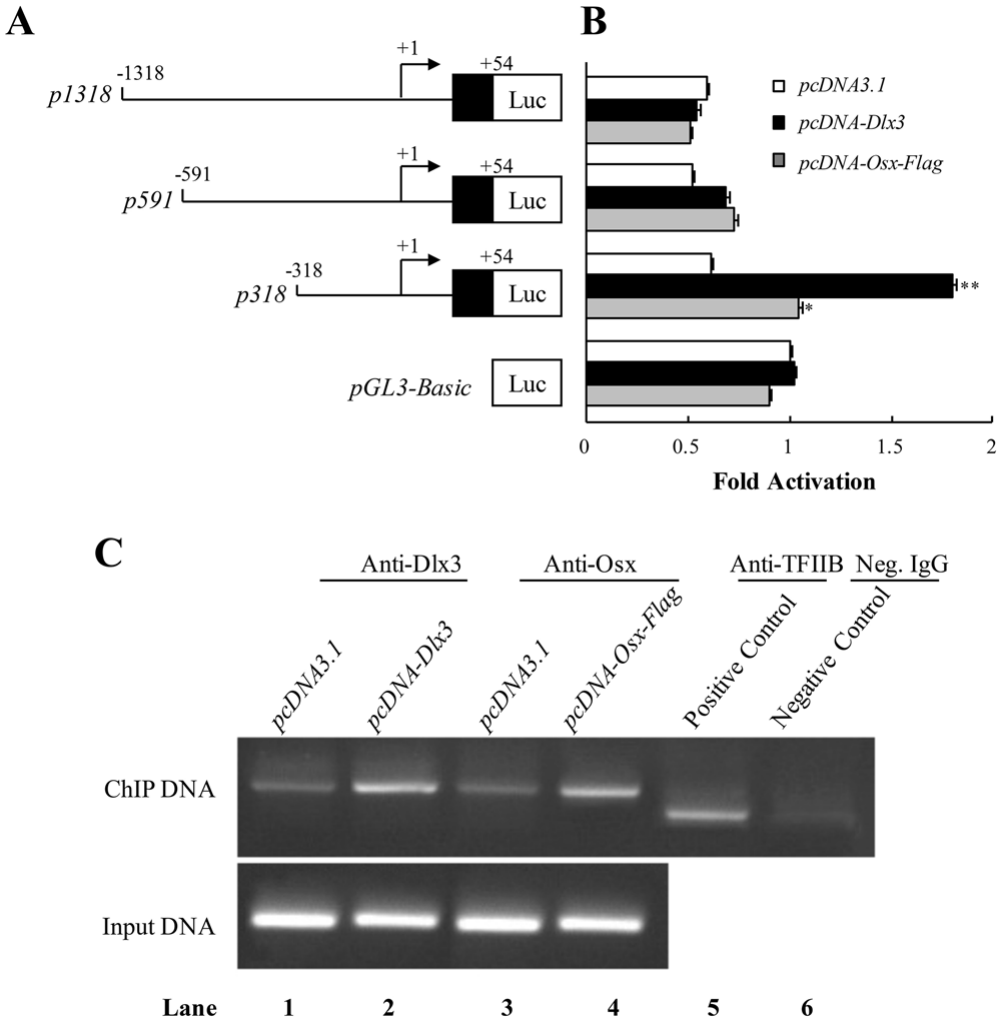
Dlx3 and Osx stimulate Dspp promoter activity. (**A**) Schematic illustration of the constructs used in the luciferase (Luc) assay. (**B**) Luciferase reporter assay showed Dlx3 and Osx enhanced *p318* promoter activity, but did not influence the activities of *p1318*, *p591* and *pGL3-Basic*. The value obtained from the untransfected control group (*pGL3-Basic* only) was taken as 1-fold, and fold increases were calculated by dividing the individual value by the control group value. *P < 0.05; **P < 0.01; compared with control group. (**C**) ChIP assay demonstrated the binding of Dlx3 and Osx with *Dspp* promoter. Neg. IgG: Negative control IgG. Uncropped images of gels were shown in Supplementary Fig. S4.

To confirm whether induction of Dsp expression by Dlx3 and Osx is through stimulating *Dspp* promoter activity, luciferase reporters containing different lengths of mouse *Dspp* promoters, *p1318*, *p591*, and *p318*, were constructed (Fig. 3A) and transiently transfected into MD10-F2 cells. Transcription activity was measured when transfected with *pcDNA-Dlx3*, or *pcDNA-Osx-Flag*, or *pcDNA3*.*1* empty vector (Fig. 3B). Both Dlx3 and Osx failed to change the transcription activity of *p1318*, *p591*, and *pGL3-Basic*, compared with transfection with *pcDNA 3*.*1* empty vector. However, overexpression of Dlx3 or Osx significantly increased the transcription activity of *p318*, which indicated both Dlx3 and Osx were able to stimulate *Dspp* promoter activity from nucleotides (nt) −318 to +54.

To further detect whether Dlx3 and Osx bind with the nt −318 to +54 of *Dspp* promoter, we performed ChIP assay. The immunoprecipitated and purified DNA was used as a template. The PCR amplifying bands with primers corresponding to the 5′-flanking region (from nt −318 to +54) of mouse *Dspp* gene were detected, and the band is more intense in cells transfected with *pcDNA-Dlx3* or *pcDNA-Osx-Flag* plasmids (Fig. 3C, lanes 1–4, upper). Positive control was done using anti-TFIIB antibody to amplify GAPDH promoter (Fig. 3C, lane 5), and negative control was set using negative control IgG (Fig. 3C, lane 6). These results indicated that Dlx3 and Osx are able to directly bind to *Dspp* promoter region from nt −318 to +54 *in vivo*.

### Identification of Dlx3 and Osx binding sites in the mouse Dspp promoter

**Figure 4:**
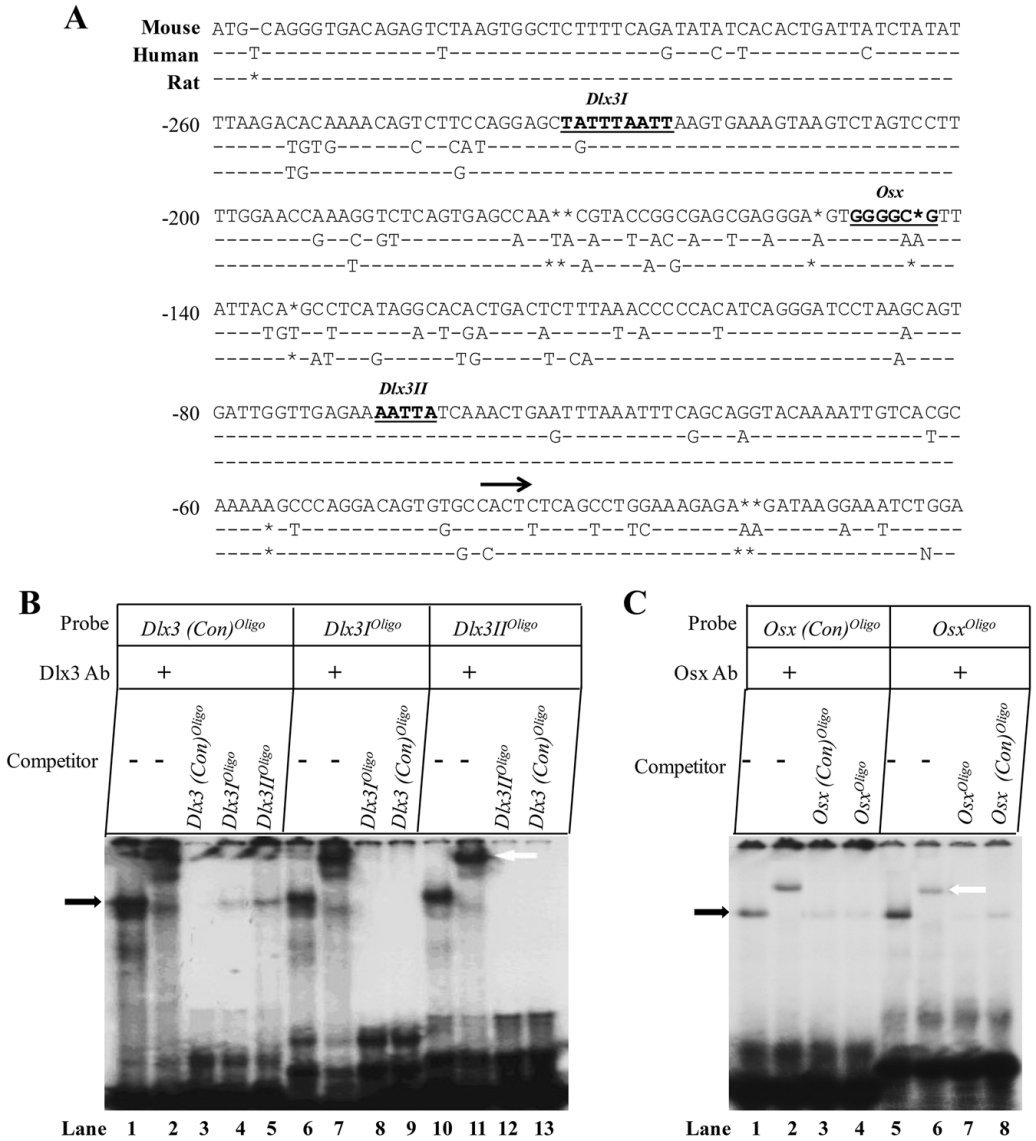
Identification of Dlx3 and Osx-binding sites in the Dspp promoter *in vitro*. (**A**) Highly homogeneity of the 5′-flanking region from −318 to +54 of the *Dspp* gene in mouse, human and rat. The putative Dlx3 and Osx binding sites are highlighted with underline. Black arrow represents the transcription start point. (**B** and **C**) EMSA was performed to determine Dlx3 and Osx binding sites in mouse *Dspp* promoter *in vitro*. Synthesized labeled oligonucleotides (Table 1) were used to incubate with recombinant Dlx3 protein (**B**) or nuclear extracts from MD10-F2 cells (**C**). The black arrows indicate protein-DNA complexes and the white arrows indicate antibody-protein-DNA complexes (Supershift bands).

It is well known Dlx3 binds to a conserved sequence of TAATT^38^, and Osx recognizes GC-rich sequence^39^. Analysis of the 5′-flanking region from nt −318 to +54 of the mouse *Dspp* gene showed two putative Dlx3 binding sites (*Dlx3I* and *Dlx3II*) and one putative *Osx* binding sites using computer software program and these sequences are highly conserved in several species, including mouse, human and rat (Fig. 4A). First, we examined whether Dlx3 and Osx could bind to their binding sites in the *Dspp* promoter using EMSA. The following oligonucleotide probes were prepared: *Dlx3I* ^*Olig*^ *°* (−232/−212) and *Dlx3II* ^*Oligo*^ (−76/−55) covering the *Dlx3I* and *Dlx3II* binding sites, *Osx* ^*Oligo*^ (−154/−132) covering the *Osx* binding site on *Dspp* promoter. After incubation, the complexes representing protein-probe binding were detected (Fig. 4B, lanes 1, 6, and 10; Fig. 4C, lanes 1 and 5, black arrow). To confirm the specificity of the complex, 100-fold unlabeled cold probes were included into the reaction and substantially competed with the binding complex (Fig. 4B, lanes 3–5, 8, 9, 12, and 13; Fig. 4C, lanes 3, 4, 7, and 8). To confirm the identity of the specifically shifted protein, anti-Dlx3 or anti-Osx antibodies were used. When the antibodies were included in the binding reaction, super-shift representing antibody-protein-probe complexes were detected (Fig. 4B, lanes 2, 7, and 11; Fig. 4C, lane 2 and 5, white arrow). These results indicated Dlx3 and Osx were able to bind to their corresponding binding sites on the *Dspp* promoter *in vitro*.

### Dlx3 and Osx depend on each other to increase *Dspp* promoter activity

**Figure 5:**
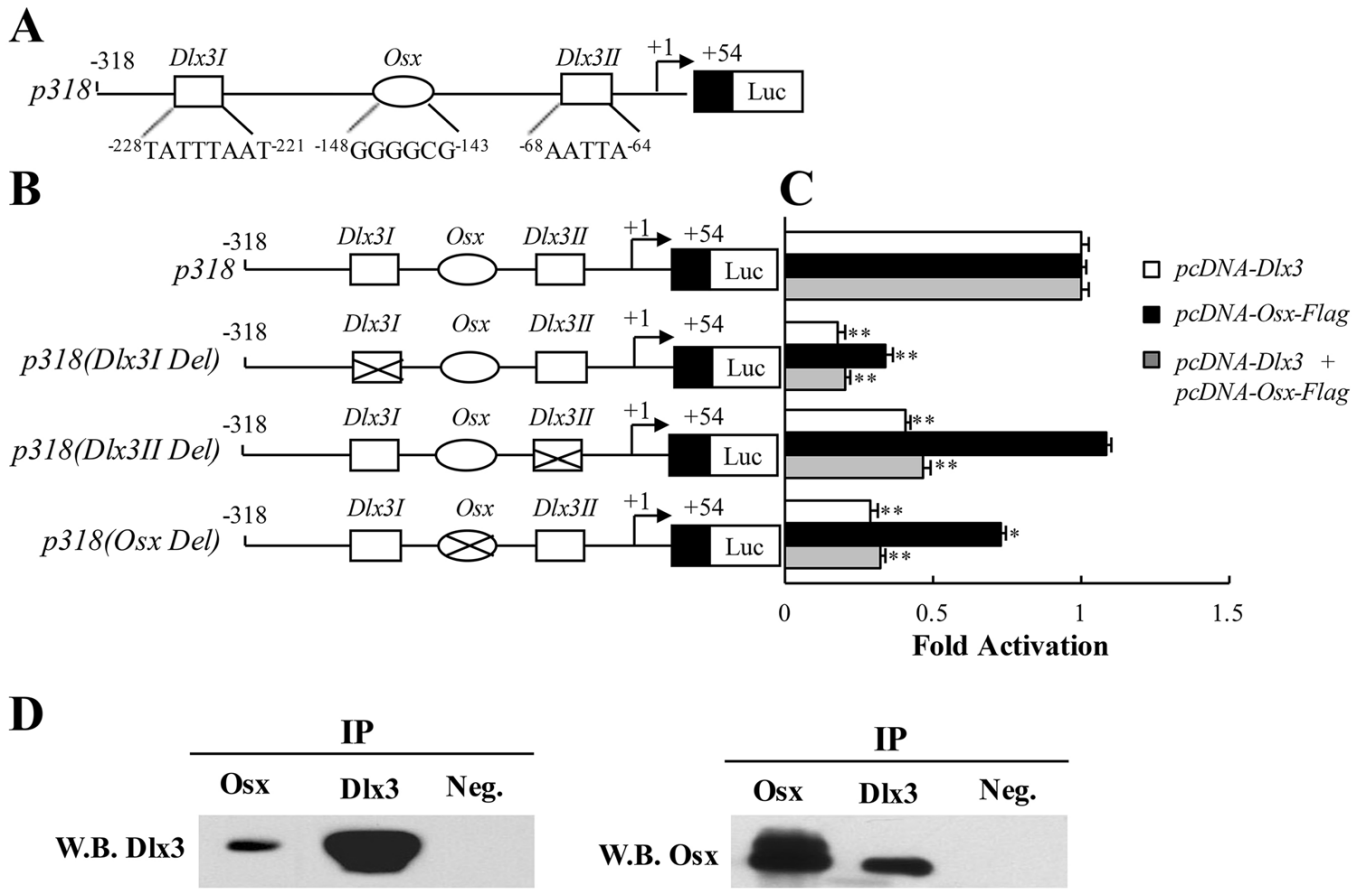
Biological activity of the Dlx3 and Osx in mouse *Dspp* promoter and protein interactions between Dlx3 and Osx. (**A**) Illustration of core sequences of *Dlx3I*, *Dlx3II* and Osx binding elements. (**B**) Illustration of wild type and mutant *p318* luciferase reporter gene constructs. The mutant region was marked with a cross. (**C**) The MD10-F2 cells were co-transfected with wild type or mutant *p318* luciferase gene constructs with *pcDNA-Dlx3* or/and *pcDNA-Osx-Flag* expression plasmids. Luciferase activity was determined, and the value obtained from the wild type *p318* group was taken as 1-fold. *P < 0.05; **P < 0.01; compared with wild type *p318* group. (**D**) Anti-Osx, anti-Dlx3 antibodies and negative control IgG were used to pull down by co-immunoprecipitation assay. Anti-Dlx3 or anti-Osx antibody was used for western blotting to confirm the presence of Dlx3 or Osx in the complex. Neg.: Negative control IgG; W.B.: Western blot. Uncropped images of blots were shown in Supplementary Fig. S5.

To further confirm the functional effect of Dlx3 and Osx binding sites in the mouse *Dspp* promoter, we generated three *p318* mutant constructs with deletion of Dlx3 or Osx binding sites (Fig. 5A and B): *p318(Dlx3I Del)*, *p318(Dlx3II Del)*, and *p318(Osx Del)*. Then *p318* or *p318 mutant* constructs were transiently transfected into MD10F-2 cells, and their transcription activity was evaluated with the co-transfection of *pcDNA-Dlx3* and/or *pcDNA-Osx-Flag*. The results showed that deletion of either *Dlx3I* or *Osx* binding site suppressed the *p318* response to both Dlx3 and Osx, but deletion of *Dlx3II* binding site only suppressed the *p318* response to Dlx3 but not to Osx (Fig. 5C). These results indicated that Dlx3 activating *Dspp* promoter requires Osx binding to its binding site on *Dspp* promoter, meanwhile Osx activating *Dspp* promoter also requires Dlx3 binding to its binding site. In particular, binding of Dlx3 to *Dlx3I* element is necessary for Osx to activate *p318 Dspp* promoter.

### Dlx3 binds to Osx forming a protein complex

The result that *Dlx3I* element is necessary for Osx activating *p318* transcription activity implied that Dlx3 may bind to Osx forming a complex to regulate *Dspp* transcription. Then, the protein-protein interaction between Dlx3 and Osx was detected with co-immunoprecipitation. As anticipated, Dlx3 was detected in the immunoprecipitation complex by Western blot with anti-Osx antibody as IP antibody (Fig. 5D, left penal). Meanwhile, Osx was also detected in the immunoprecipitation complex with anti-Dlx3 antibody as IP antibody (Fig. 5D, right penal), which indicated Dlx3 bound to Osx forming protein complex.

## Discussion

Odontoblast differentiation is regulated by many transcription and growth factors. BMP-2 signaling induces dental mesenchymal cell differentiation into odontoblasts^40^. *Dspp* has been characterized as a unique marker of odontoblasts differentiation^1, 2^. It has been reported that BMP-2 is able to induce *Dspp* expression, but up to now, the molecular mechanisms by which BMP-2 induces *Dspp* transcription have not been well elucidated. The present investigation reveals a new signaling pathway, which participates in the regulation of *Dspp* expression by BMP-2 in odontoblasts.

Dlx3 contains a homeodomain, which is related to the distal-less domain of *Drosophila* and was detected even in structures involving epithelial-mesenchymal interaction, such as tooth germs and hair follicles. BMP-2 was able to induce *Dlx3* gene expression in several tissue cells, including osteoblasts^27^, keratinocytes^41^, and dental follicle cells^42^. In addition to *Dlx3*, two other members of the *distal-less* family, *Dlx2* and *Dlx5*, are also regulated by BMP-2 in osteogenic or chondrogenic cells^27, 43^. Osx as a transcription factor was first identified in C2C12 myogenic cells treated with BMP-2^39^. Besides,it has also been reported that Osx was induced with BMP-2 in mouse progenitor cells and chondrocytes^39, 44^, human and mouse mesenchymal stem cells^45, 46^. Consistent with these previous investigations, we also demonstrated that Dlx3 and Osx were BMP-2-inducable in odontoblasts both *in vitro* and *in vivo*. Previously, an Osx-GFP fusing protein reporter system was constructed to track Osx translocation in osteogenic cells^47^. Osx remained mostly inactive in the cytosol of non-osteogenic cells and was activated and translocated into the nucleus with the osteogenic differentiation of osteogenic cells^47^. In the present investigation, as showed by immunofluorescence staining, we found both Dlx3 and Osx were activated and translocated into nucleus from cytoplasm with addition of BMP-2 *in vitro* and *in vivo*.

Although *Osx* mRNA expression is stimulated by BMP-2 treatment, pretreatment with cycloheximide, a protein synthesis inhibitor, blocks the BMP-2-induced expression of *Osx* mRNA^37^, which indicates that *Osx* is not the direct target of the BMP signaling cascade and the expression of *Osx* induced by BMP-2 requires the intermediation of newly synthesized proteins. In contrast to *Osx* expression induced by BMP-2, BMP-2-induced *Dlx5* expression is unaffected by cycloheximide pretreatment^48, 49^, which means *Dlx5* is a direct target of BMP signaling. Moreover, inhibition of Dlx5 expression blocked Osx expression^50^, indicating Osx is down stream target of Dlx5. In the present investigation, we found the similar results: overexpression of Dlx3 was able to induce Osx expression, but overexpression of Osx did not influence the expression of Dlx3. Furthermore, down-regulated Osx expression in cell with transfection of *Dlx3*-targeted siRNA further confirmed Osx is a downstream target of Dlx3 in odontoblasts. The fact that the time of Dlx3 to reach the maximum level after BMP-2 treatment is earlier than that of Osx indicates that Osx is also not the direct target of the BMP-2 in odontoblasts.

Both positive and negative regulatory mechanisms are required for the spatial and temporal expression of *Dspp* gene. Many investigations characterized mouse and rat *Dspp* gene promoters and found inverted TATA and CAATT box sequences, Sp1, Nrf1, C/EBP and Runx2 binding sites, as well as several homeodomain (Dlx and Msx) motifs in the proximal regions^8, 51–53^. Previously, we found BMP-2 regulates *Dspp* expression through the activation of the heterotrimeric transcription factor NF-Y, and NF-Y binds to a BMP-2 response element in the mouse *Dspp* promoter, especially between nt −97 and −72^3^. In the present investigation, we found that in addition to NF-Y, BMP-2 also regulates *Dspp* expression also through the Dlx3 and Osx binding sites on *Dspp* promoter. Two Dlx3 binding sites (*DLX3I*, *DLX3II*) and one Osx binding site were identified in the proximal mouse *Dspp* promoter that mediates BMP-2-stimulated *Dspp* expression. EMSA and ChIP analyses verified that Dlx3 and Osx bind to their target sites in the mouse *Dspp* promoter. To further evaluated the function of these binding sequences, direct deletion in the *Dspp* promoter was used, and showed that deletion of either Dlx3 or Osx binding site decreased *Dspp* promoter activity in MD10-F2 cells, which means each Dlx3 or Osx binding site in the *Dspp* promoter was functional. Duverger *et al*.^33^ demonstrated the direct binding of Dlx3 to *Dspp* promoter, which is in consistent with our findings. In *distal-less* family, Dlx5 also mediates *Dspp* expression regulated by BMP signal^22^. Although both Dlx3 and Dlx5 can bind to TAAT box, but the two Dlx3 binding sites found in the present research only respond to Dlx3 but not to Dlx5^22^, which indicates Dlx3 and Dlx5, as distal-less family members, show different mechanism in regulation of *Dspp* transcription. Both Dlx3 and Osx can increase the promoter activity of *p318*, but not *p591* and *p1318*. One possibility is that there are Dlx3 and Osx binding sites at upstream of nt −318 on *Dspp* promoter, but Dlx3 and Osx act as suppressors when binding to these binding sites. Another possibility is that there are strong negative regulatory elements for the binding of other transcription factors at upstream of nt −318 on *Dspp* promoter, and Dlx3 and Osx as co-factors could increase the suppressing activity of these elements. Cao *et al*.^22^ found that deletion of the region from −791 to −427 strongly increased basal *Dspp* promoter activity, and inferred there might be a strong negative regulatory element between nt −794 and −427. Narayanan *et al*.^8^ also identified a repression domain between nt −700 and −400 of *Dspp* promoter. Further analysis of this region identified two binding elements, which have 90% homology to the DNA binding sites for two bZIP transcription factors, Nrf1 and the CCAAT enhancer-binding protein (C/EBP) β. A possible response element for Yy1, a negative transcription factor, was also found (at position of nt −622) in this repression region of *Dspp* promoter, and the basal promoter activity of *Dspp* increased after the Yy1 binding sites was deleted^22^.

By means of co-immunoprecipitation assay we have clearly demonstrated that Dlx3 associates and functionally cooperates with Osx during regulation of *Dspp* transcription. Mutational studies suggest that the Dlx3 and Osx depend on each other to increase *Dspp* promoter activity. Dlx3 could not activate the promoter activity by binding itself to the *Dspp* promoter containing the mutated Osx binding site. All these results indicate the bindings of Dlx3 and Osx to their binding sites on *Dspp* promoter are necessary for each of these two transcription factors to activate *Dspp* promoter.

In conclusion, in this study we provide the evidence that BMP-2 activates *Dspp* gene transcription via Dlx3/Osx signaling pathway: BMP-2 interacting with its receptor on cell membrane induces Dlx3 expression, then Dlx3 induces Osx expression; subsequently, both Dlx3 and Osx are activated, enter nucleus and bind to their target sites in *Dspp* promoter; then Dlx3 interacts with Osx and synergistically stimulate *Dspp* transcription.

## Methods

### Animals and Tissue Preparation

All experimental procedures involving the use of animals were reviewed and approved by the Institutional Animal Care at the University of Texas Health Science Center at San Antonio. All experiments were performed in accordance with the relevant guidelines and regulations. The generations of *Bmp2* ^*f/f*^ and *3*.*6Col1a1-Cre* mice have been described previously^54, 55^. To conditionally knock out *Bmp2* gene in odontoblasts, *3*.*6Col1a1-Cre;Bmp2* ^*f/f*^ (referred to as *Bmp2-cKO* ^*od*^) mice were generated by crossing *3*.*6Col1a1-Cre;Bmp2* ^*f/+*^ allele with *Bmp2* ^*f/f*^ allele. Mice at postnatal day 3 (P3) were put to death. Mandibles of the mice were immediately dissected and fixed with 4% paraformaldehyde (PFA) for 24 hours at 4 °C. After washes with PBS, samples were demineralized in 8% EDTA for 2 weeks. Then, the tissues were processed for paraffin embedding and sectioned at 5 µm.

### Cell Culture and Treatment

A mouse immortalized preodontoblast cell line, MD10-F2 was used^56^. Cells were grown at 33 °C under 5% CO_2_ in alpha minimum essential medium (α-MEM) supplemented with 10% fetal bovine serum, 100 units/ml penicillin/streptomycin, 50 µg/ml ascorbic acid and 10 mM sodium β-glycerophosphate. For BMP-2 induction, cells were starved overnight in FBS-free medium, then cells were induced with 100 ng/ml of recombinant human BMP-2 protein (R&D) for the indicated times. Then the cells were subjected to Western blot or immunofluorescence staining.

### Immunohistochemistry and Immunofluorescence Staining

The expressions of Dlx3 and Osx in mandibular first molars of both wild type and *Bmp2-cKO* ^*od*^ mice were analyzed by immunohistochemistry as described previously^57^. MD10-F2 cells cultured on glass slides were fixed with cold methanol/acetone (1:1) for 10 minutes and permeabilized with 0.5% Triton-X for 15 minutes. To block the non-specific binding of antibodies, slides were incubated with 10% goat serum for 30 minutes, followed by primary antibodies, anti-Dlx3 (Abcam) and anti-Osx (Santa Cruz), overnight at 4^o^C. After washing with PBS, slides were incubated with secondary IgG antibodies conjugated to Alexa-Fluor 568 (Invitrogen) were added and incubated for 1 hour. Hoechst (Pierce) was used to stain nucleus.

### Western Blot

Western blot was performed with whole cell lysates from MD10-F2 cells. Cells were washed with cold PBS and lysed with RIPA buffer (1× PBS, 1% Nonidet P-40, 0.5% sodium deoxycholate, 0.1% SDS, 10 mg/ml phenylmethylsulfonyl fluoride, 30 μl/ml aprotinin, 100 mM sodium orthovanadate). Proteins (40 μg/well) were resolved by 10% SDS-PAGE and transferred to a Trans-blot membrane (Bio-Rad). Western blot was performed as described earlier^2^. Anti-Dlx3, anti-Osx, and anti-Dsp (Santa Cruz) was used as primary antibodies. β-actin antibody (Santa Cruz) was used as a loading control.

### Construction of Reporter Gene Constructs, Expression Plasmids, and Mutagenesis

All reporter gene constructs containing 5′ deletions of the *Dspp* promoter were generated using standard cloning procedures as described previously^58^. Briefly, the 1372-bp BglII-HindIII fragment of the mouse *Dspp* gene from nucleotides (nt) −1318 to +54 was cloned into the BglII and HindIII sites of *pGL3-Basi*c luciferase vector (Promega) and designated *p1318*. The 646-bp EcoRI-HindIII fragment of the mouse *Dspp* gene from nt −591 to +54 was cloned into the EcoRI and HindIII sites of the *pGL3-Basic* and called *p591*. The 372-bp XhoI-HindIII fragment of the mouse *Dspp* gene from nt −318 to +54 was cloned into the XhoI and HindIII sites of *pGL3-Basic* vector and called *p318*. All these various plasmids contained part of the exon 1 noncoding region of *Dspp* gene.

For the generation of expression constructs, the full length of *Dlx3* cDNA or *Osx* cDNA containing a *Flag* tag was subcloned into *pcDNA 3*.*1* vector (*pcDNA-Dlx3*, *pcDNA-Osx-Flag*). All constructs were confirmed by DNA sequencing.

Site directed deletion targeting the Dlx3 and Osx binding sites in the *Dspp* promoter was performed using the Quickchange® II Site-Directed Mutagenesis Kit (Stratagene). Mutant plasmids, *p318(Dlx3I Del)*, *p318(Dlx3II Del)*, and *p318(Osx Del)*, were generated by site-directed deletion at −228/−221, −68/−64, and −148/−143 sites of p318 construct as a template. Primers for mutant plasmids were as follows: *p318(Dlx3I Del)*, 5′-CTTCCAGGAGCAAGTGAAAGTAAGTCTAGTCC-3′, and 5′-GGACTAGACTTACTTTCACTTGCTCCTGGAAG-3′; *p318 (Dlx3II Del)*, 5′-GCAGTGATTGGTTGAGAATCAAACTGAATTTAAATTTCAGC-3′, and 5′-GCTGAAATTTAAATTCAGTTTGATTCTCAACCAATC ACTGC-3′; *p318(Osx Del)*, 5′-GCGAGCGAGGGAGTCGTTATTACAGCCTC-3′, and 5′-GAGGCTGTAATAACGACTC CCTCGCTCGC-3′. All constructs were checked by DNA sequencing and purified using Plasmid Midi Kit (Qiagen).

### Overexpression and RNA Interference

MD10-F2 cells were transfected with *pcDNA-Dlx3* or *pcDNA-Osx-Flag* or empty vector using Lipofectamine 2000 transfection reagent (Invitrogen). To knock down the endogenous Dlx3 expression, small interfering RNA (siRNA) targeted mouse *Dlx3* or negative control siRNA (Santa Cruz) were transfected into MD10-F2 cells at 60 nM concentration with Lipofectamine 2000. Cell lysates were collected 24 or 48 hours after transfection and protein levels of Dlx3, Osx and Dsp were analyzed by Western blot.

### Luciferase reporter assay

Cell were seeded in 48-well plates and cultured overnight. Then wild-type or mutant reporter plasmids or empty *pGL3-Basic* plasmid and *pRL-TK* Renilla luciferase reporter (Promega), as well as *pcDNA-Dlx3* or/and *pcDNA-Osx-Flag* were co-transfected into cells using the Lipofectamine 2000 for 48 hours. After that, the cells were collected and lysed in passive lysis buffer (Promega). The luciferase assay was performed using the Dual Luciferase Reporter Assay System (Promega) according to the manufacture’s protocols. Firefly and Renilla luciferase activities were qualified using the Glomax Luminometer (Promega). Firefly luciferase activities was normalized against Renilla luciferase activities. All luciferase assays were performed in triplicate at least three times.

### Chromatin Immunoprecipitation (ChIP)

ChIP were performed according to the instructions provided by ChIP-IT^TM^ kit (Active Motif). Briefly, MD10-F2 cells were co-transfected with *pcDNA-Dlx3*, *pcDNA-Osx-Flag* expression plasmids and *p318* plasmid. After 72 hours of transfection, cells were washed in PBS and incubated for 10 minutes with 1% formaldehyde. After quenching the reaction with 0.1 M glycine, the cross-linked material was sonicated into chromatin fragment of an average length of 200–800 bp. After pre-cleaning, 1% of each sample was saved as input fraction. The chromatin solution was precleared by adding protein G beads with salmon sperm DNA for 2 hours at 4 °C. Then immunoprecipitations were performed with protein G beads and 5 μg of anti-Dlx3 or Osx antibodies overnight at 4 °C. Anti-TFIIB antibody and negative control IgG (all from Santa Cruz Biotechnology) were used as positive and negative controls. Immunoprecipitated materials were washed, and cross-links were reversed by incubating samples for 5 hours at 65 °C in 200 mM NaCl and 10 μg of Rnase A to eliminate RNA. Recovered material was treated with proteinase K, and the DNA fragment was purified by Qiagen columns (Qiagen). The purified DNA was analyzed by PCR amplifying promoter regions (−318/+54) of the *Dspp* gene using the following primer pairs: forward ^−318^5′-GAAATGCAGGGTGACAGAGTCTAAGTGGCT-3′^−289^, and reverse^+54^5′-CGAGGGG ACTTTGAAAATCCAGATT-3′^+30^. The sequence of positive control PCR primers was provided by the kit, which flank the GAPDH promoter. The sequence of negative control PCR primers was also provided by the kit, which flank a region of genomic DNA between the GAPDH gene and the chromosome condensation-related SMC-associated protein (CNAP1) gene.

### Electrophoretic Mobility Shift Assay (EMSA)

Recombinant Dlx3 protein was purchased from Novus Biologicals. Nuclear extracts from MD 10-F2 cells were prepared using the method of Dignam *et al*.^59^. Protein concentration was determined by using the Bradford assay^60^. Oligonucleotides used in EMSA were synthesized as listed in Table 1. *Dlx3 (Con)* ^*Oligo*^ and *Osx (Con)* ^*Oligo*^ were set as positive control, which were verified containing two copies of the Dlx3 binding sites and one GC-rich Osx binding site, respectively^39, 61^. *Dlx3I* ^*Oligo*^, *Dlx3II* ^*Oligo*^, and *Osx* ^*Oligo*^ consist of the putative Dlx3 or Osx binding region of *Dspp* promoter. These oligonucleotides were labeled with [γ-^32^P]ATP. Double-stranded probes were generated by annealing [γ-^32^P]ATP—labeled complementary oligonucleotides. EMSA was performed as described previously^58^. For the competition binding reactions, the unlabeled competitor in 100-fold molar excesses of the labeled probe was included in the reaction. Antibody super-shift experiments were performed with anti-Dlx3 and anti-Osx antibodies. The antibodies were added to recombinant Dlx3 protein or the nuclear extracts 10 min prior to the addition of the radiolabeled prob. Free and protein-bound DNA complexes were loaded onto a 5% native polyacrylamide gel in 1 × Tris/boric acid/EDTA (TBE) buffer, electrophoresed, dried, and exposed to X-ray film.

**Table 1:** EMSA and competitions were performed with the oligonucleotide duplexes listed below

Name	Sequence (5′ to 3′)
*Dlx3 (Con)* ^*Oligo*^	5′-GCGATAATTGCGATAATTGCGAAG-3′
*Dlx3I* ^*Oligo*^	^−232^5′-GAGCTATTTAATTAAGTGAAA-3′^−212^
*Dlx3II* ^*Oligo*^	^−76^5′-GTTGAGAAAATTATCAAACTGA-3′^−55^
*Osx (Con)* ^*Oligo*^	5′-GCTCGCCCCGCCCCGATCTGAAT-3′
*Osx* ^*Oligo*^	^−154^5′-GGGAGTGGGGCGTTATTACAGCC-3′^−132^

*The core binding sequence was underlined*.

### Co-immunoprecipitation (Co-IP) Analysis

MD10-F2 cells cultured in 10-cm dish were transfected with 10 μg of *pcDNA-Dlx3* and *pcDNA-Osx-Flag*. After 72 hours of transfection, cells were lysed with RIPA lysis buffer, and centrifuged at 12,000 rpm at 4 °C for 20 minutes. The supernatant was collected and precleared with 40 μl of protein A/G plus agarose beads (Santa Cruz) at 4 °C for 30 minutes. Immunoprecipitation was performed with anti-Dlx3, anti-Osx, anti-Flag (Sigma) antibodies or negative control IgG (3 μg each) and immunocomplexes were precipitated with 50 μl of protein A/G plus agarose beads. Beads were washed three times with wash buffer (10 mM Tris-HCl, pH 8.0, 150 mM NaCl, 10% glycerol, 1% NP-40, and 2 mM EDTA). Protein was eluted by boiling in 50 μl of sample buffer for 10 minutes and analyzed by Western blot.

## Statistical Analysis

Quantitative data were presented as means ± SD from three independent experiments and analyzed with Analysis of Variance (ANOVA) or Student’s *t* test with SPSS. For Western blot, densitometry of immunoblot bands were analyzed with ImageJ software.

## Electronic supplementary material

Supplementary data
