# Supplementary material for: BMP-2 induced Dspp transcription is mediated by Dlx3/Osx signaling pathway in odontoblasts
Source: Sci Rep. 2017 Sep 7;7:10775. doi: 10.1038/s41598-017-10908-8 (PMC5589848; doi:10.1038/s41598-017-10908-8)
Supplement: Supplementary file 1 — Supplementary data [file 41598_2017_10908_MOESM1_ESM.pdf]

Supplementary Information

Title:

BMP-2 induced *Dspp* transcription is mediated by Dlx3/Osx signaling pathway in odontoblasts

Author list:

Guobin Yang, Guohua Yuan, Mary MacDougall, Chen Zhi, Shuo Chen

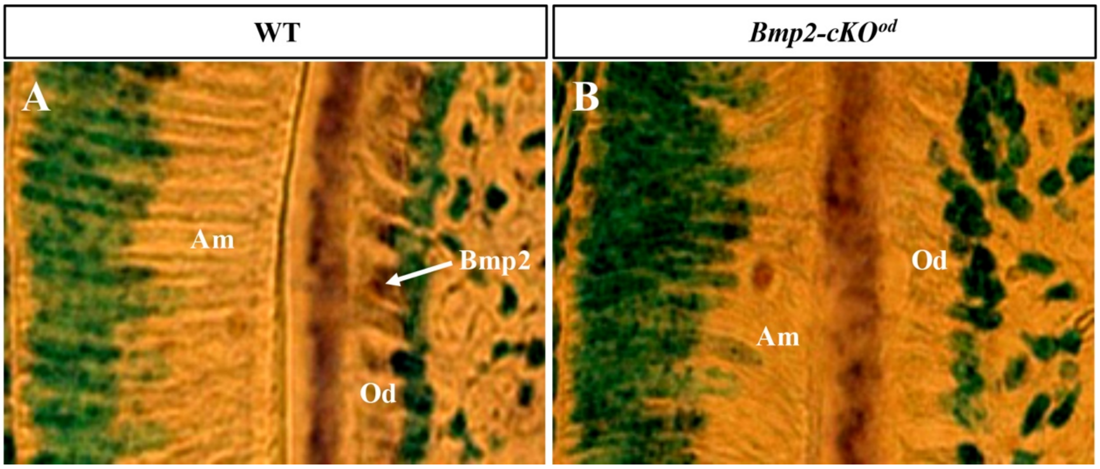

Supplementary Figure S1. *In situ* hybridization of *Bmp2* mRNA in first molar of 12-day old mice. A: wild type mice; B: *Bmp2-cKO*^{od} mice. Blue-purple color represents the hybridization signal. Am: Ameloblasts; Od: Odontoblasts.

B

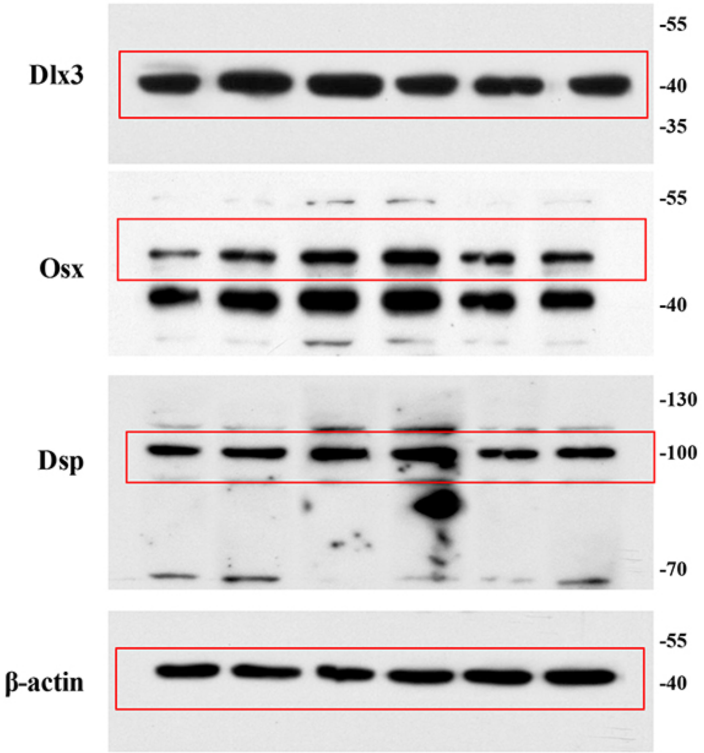

Supplementary Figure S2. Uncropped images of bots presented in the main Figure 1.

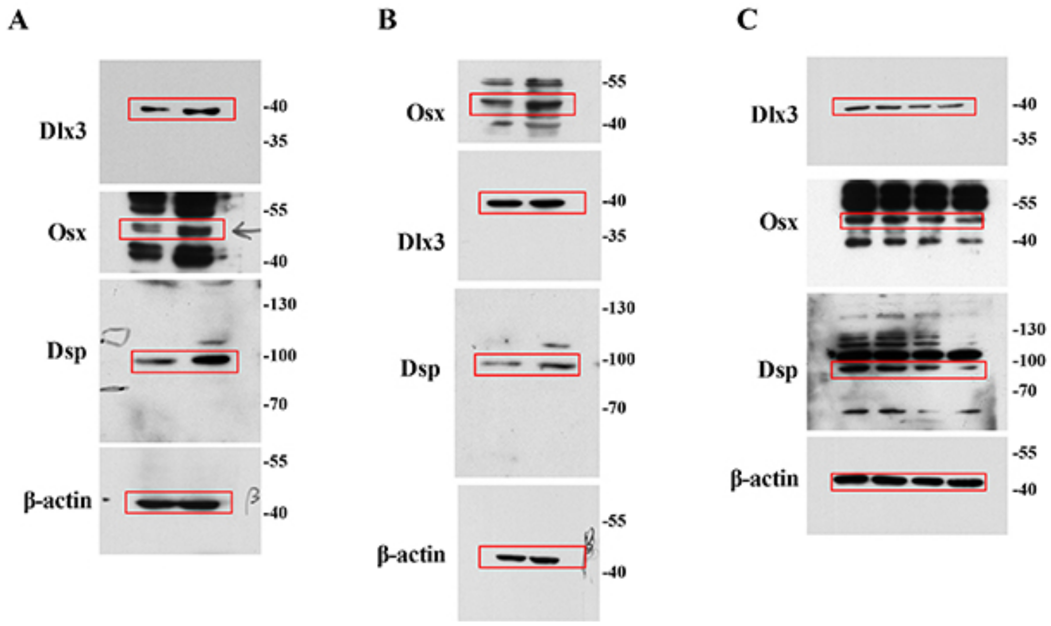

Supplementary Figure S3. Uncropped images of bots presented in the main Figure 2.

C

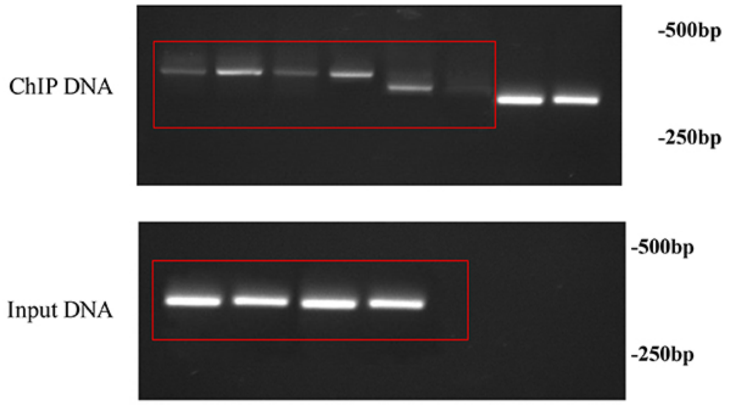

Supplementary Figure S4. Uncropped images of gels presented in the main Figure 3.

D

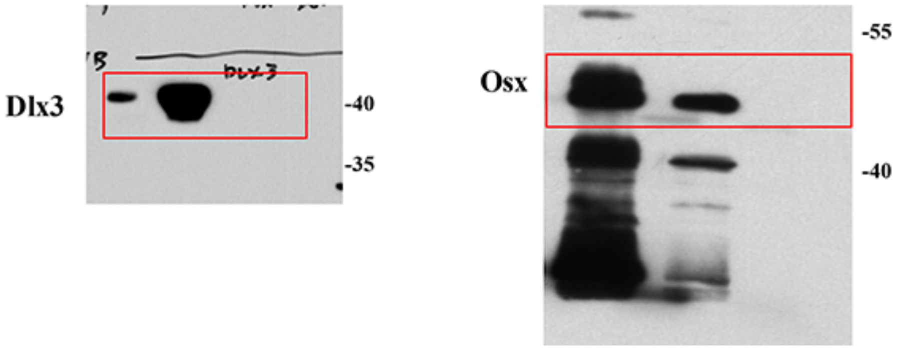

Supplementary Figure S5. Uncropped images of bots presented in the main Figure 5.
